# Crystal structure of 4-[1-(2-hy­droxy­prop­yl)-4,5-diphenyl-1*H*-imidazol-2-yl]benzoic acid

**DOI:** 10.1107/S2056989014027078

**Published:** 2015-01-03

**Authors:** Jerry P. Jasinski, Shaaban K. Mohamed, Mehmet Akkurt, Antar A. Abdelhamid, Mustafa R. Albayati

**Affiliations:** a Department of Chemistry, Keene State College, 229 Main Street, Keene, NH 03435-2001, USA; b Chemistry and Environmental Division, Manchester Metropolitan University, Manchester M1 5GD, England; c Chemistry Department, Faculty of Science, Minia University, 61519 El-Minia, Egypt; d Department of Physics, Faculty of Sciences, Erciyes University, 38039 Kayseri, Turkey; e Chemistry Department, Faculty of Science, Sohag University, Sohag, Egypt; f Kirkuk University, College of Science, Department of Chemistry, Kirkuk, Iraq

**Keywords:** crystal structure, 4-[1-(2-hy­droxy­prop­yl)-4,5-diphenyl-1*H*-imidazol-2-yl]benzoic acid, imidazole ring, amino alcohol

## Abstract

In the title compound, C_25_H_22_N_2_O_3_, the central imidazole ring makes dihedral angles of 48.43 (10), 20.23 (10) and 75.38 (11)° with the benzene ring and the two phenyl rings, respectively. The phenyl ring adjacent to the N-bonded 2-hy­droxy­propyl group shows the greatest twist, presumably to minimize steric inter­actions. In the crystal, mol­ecules are linked by O—H⋯N, O—H⋯O and C—H⋯O hydrogen bonds, forming a three-dimensional network. In addition, C—H⋯π inter­actions are also observed.

## Related literature   

For similar structures and background to the biological properties of imidazole derivatives, see: Akkurt *et al.* (2013 ▸); Mohamed *et al.* (2013*a* ▸,*b* ▸). For the synthesis of the title compound, see: Mohamed *et al.* (2012 ▸).
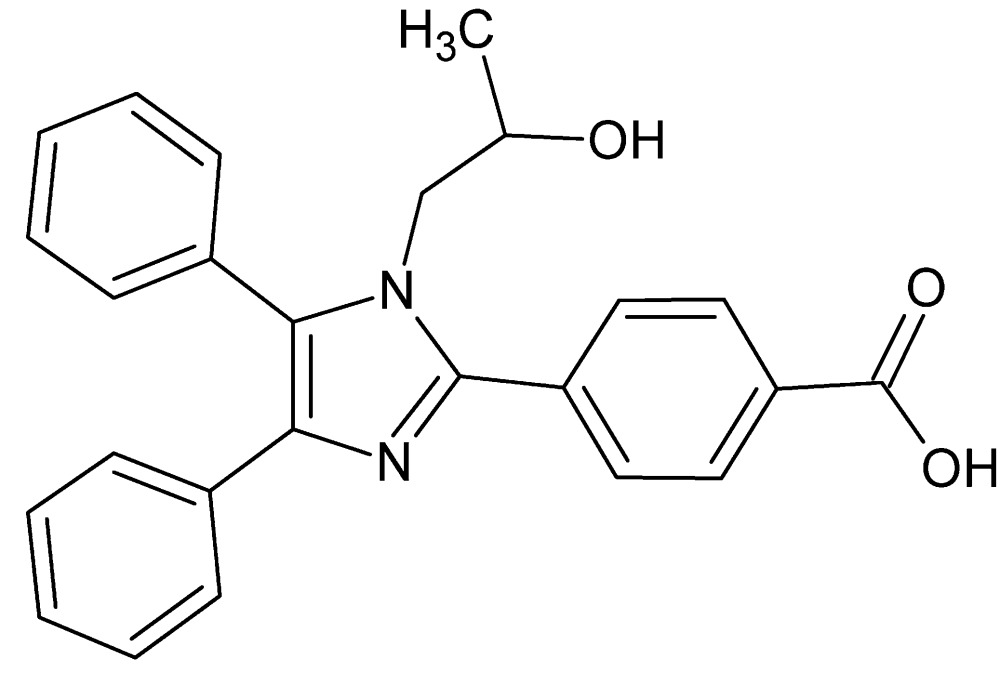


## Experimental   

### Crystal data  

C_25_H_22_N_2_O_3_
*M* *_r_* = 398.45
Triclinic, 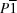

*a* = 6.8710 (4) Å
*b* = 10.7188 (6) Å
*c* = 14.9178 (7) Å
α = 103.569 (4)°
β = 93.094 (4)°
γ = 105.878 (5)°
*V* = 1019.03 (10) Å^3^
*Z* = 2
Cu *K*α radiation
μ = 0.69 mm^−1^
*T* = 293 K
0.36 × 0.32 × 0.24 mm

### Data collection  

Agilent Xcalibur (Eos, Gemini) diffractometer
Absorption correction: multi-scan (*CrysAlis PRO*; Agilent, 2014 ▸) *T*_min_ = 0.928, *T*_max_ = 1.000
6591 measured reflections
3859 independent reflections
3209 reflections with *I* > 2σ(*I*)
*R*_int_ = 0.022

### Refinement  

*R*[*F* ^2^ > 2σ(*F* ^2^)] = 0.048
*wR*(*F* ^2^) = 0.136
*S* = 1.02
3859 reflections
274 parameters
H-atom parameters constrained
Δρ_max_ = 0.25 e Å^−3^
Δρ_min_ = −0.25 e Å^−3^

### 

Data collection: *CrysAlis PRO* (Agilent, 2014 ▸); cell refinement: *CrysAlis PRO*; data reduction: *CrysAlis PRO*; program(s) used to solve structure: *SHELXS2013* (Sheldrick, 2008 ▸); program(s) used to refine structure: *SHELXL2013* (Sheldrick, 2008 ▸); molecular graphics: *ORTEP-3 for Windows* (Farrugia, 2012 ▸); software used to prepare material for publication: *PLATON* (Spek, 2009 ▸).

## Supplementary Material

Crystal structure: contains datablock(s) global, I. DOI: 10.1107/S2056989014027078/hb7338sup1.cif

Structure factors: contains datablock(s) I. DOI: 10.1107/S2056989014027078/hb7338Isup2.hkl

Supporting information file. DOI: 10.1107/S2056989014027078/hb7338Isup3.cml

**Fig. 1.**
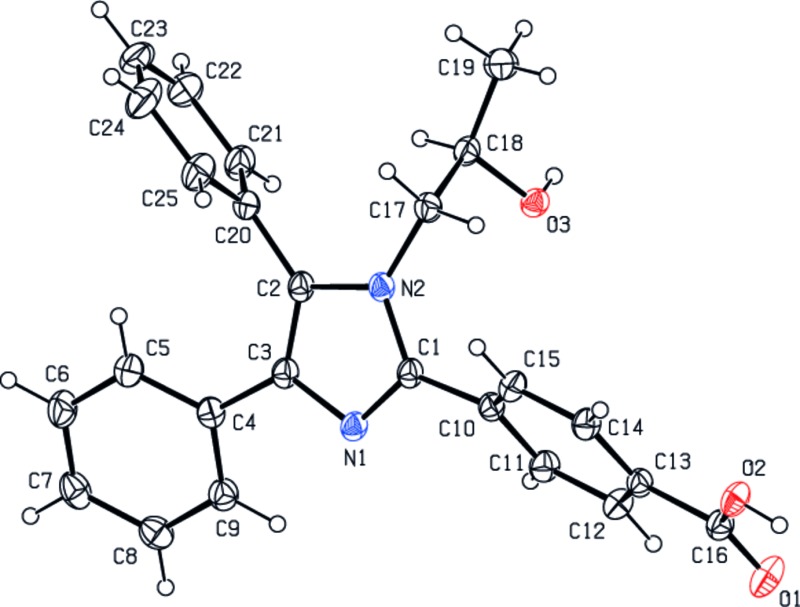
Perspective view of the title molecule with 50% probability ellipsoids.

. DOI: 10.1107/S2056989014027078/hb7338fig1.tif
Perspective view of the title mol­ecule with 50% probability ellipsoids.

**Fig. 2.**
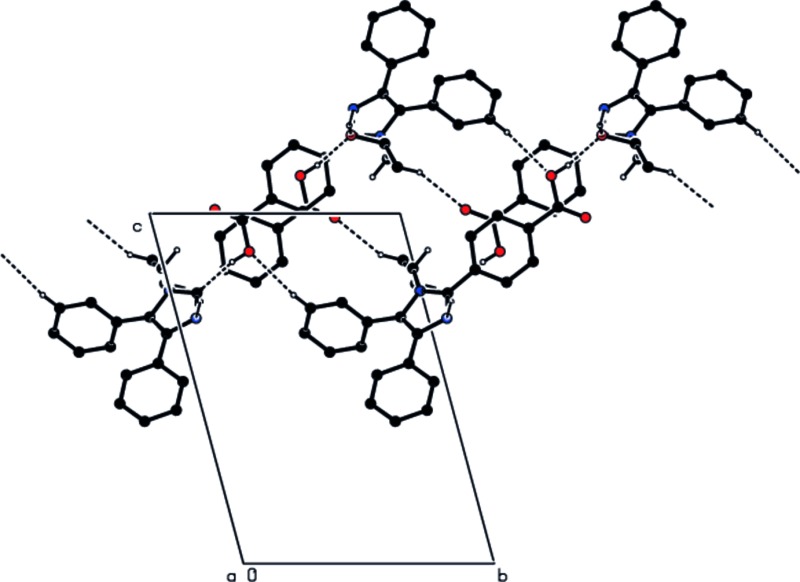
View of a part of the hydrogen bonding in the title compound

. DOI: 10.1107/S2056989014027078/hb7338fig2.tif
View of a part of the hydrogen bonding in the title compound

CCDC reference: 1038591

Additional supporting information:  crystallographic information; 3D view; checkCIF report

## Figures and Tables

**Table 1:** Hydrogen-bond geometry (, ) *Cg*1 is the centroid of the N1/N2/C1C3 ring.

*D*H*A*	*D*H	H*A*	*D* *A*	*D*H*A*
O2H2O3^i^	0.82	1.86	2.6718(17)	170
O3H3N1^ii^	0.82	2.04	2.8377(19)	166
C19H19*B*O1^iii^	0.96	2.46	3.355(3)	155
C24H24O2^iv^	0.93	2.57	3.482(3)	167
C19H19*C* *Cg*1^ii^	0.96	2.53	3.422(2)	154

Symmetry codes: (i) 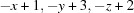; (ii) 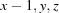; (iii) 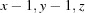; (iv) 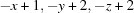.

## supplementary crystallographic information

### S1. Comment

Following to our ongoing study on synthesis of imidazole based amino alcohols (Akkurt *et al.*, 2013; Mohamed *et al.*, 2013*a*,b) we herein report the synthesis and crystal structure of the title compound.

In the title compound, Fig. 1, the central 1*H*-imidazole ring (N1/N2/C1—C3) makes dihedral angles of 48.43 (10), 20.23 (10) and 75.38 (11)°, with the benzene ring (C10–C15) and two phenyl rings (C4–C9 and C20–C25), respectively. The dihedral angle between the (C4–C9 and C20–C25) phenyl rings is 69.94 (11)°. The (C10–C15) benzene ring forms dihedral angles of 36.05 (10) and 35.91 (10)° with two the phenyl rings (C4–C9 and C20–C25), respectively. The bond lengths are comparable to those reported for similar compounds reported in the literature (Akkurt *et al.*, 2013; Mohamed *et al.*, 2013*a*,b).

In the crystal, O—H···N, O—H···O and C—H···O hydrogen bonds link the adjacent molecules, into a three dimensional network structure (Table 1, Fig. 2). Furthermore, C—H···π interactions (Table 1) are also observed in the packing of the title compound.

### S2. Experimental

The title compound has been prepared according to our reported method (Mohamed *et al.*, 2012). Irregular colourless chunks of (I) were obtained by the slow evaporation method using ethanol as a solvent. *M*.p. 456 K.

### S3. Refinement

All hydrogen atoms were located in geometrically idealized positions and constrained to ride on their parent atoms, with C—H = 0.93–0.98 Å, O—H = 0.82 Å and *U*_iso_(H) = 1.2*U*_eq_(C) or *U*_iso_(H) = 1.5*U*_eq_(O).

### Crystal data

**Table:**

C_25_H_22_N_2_O_3_	*Z* = 2
*M**_r_* = 398.45	*F*(000) = 420
Triclinic, *P*1	*D*_x_ = 1.299 Mg m^−^^3^
Hall symbol: -P 1	Cu *K*α radiation, λ = 1.54184 Å
*a* = 6.8710 (4) Å	Cell parameters from 2716 reflections
*b* = 10.7188 (6) Å	θ = 4.7–70.9°
*c* = 14.9178 (7) Å	µ = 0.69 mm^−^^1^
α = 103.569 (4)°	*T* = 293 K
β = 93.094 (4)°	Irregular, colourless
γ = 105.878 (5)°	0.36 × 0.32 × 0.24 mm
*V* = 1019.03 (10) Å^3^	

### Data collection

**Table:**

Agilent Xcalibur (Eos, Gemini) diffractometer	3859 independent reflections
Radiation source: Enhance (Cu) X-ray Source	3209 reflections with *I* > 2σ(*I*)
Graphite monochromator	*R*_int_ = 0.022
Detector resolution: 16.0416 pixels mm^-1^	θ_max_ = 71.3°, θ_min_ = 3.1°
ω scans	*h* = −8→8
Absorption correction: multi-scan (*CrysAlis PRO*; Agilent, 2014)	*k* = −13→11
*T*_min_ = 0.928, *T*_max_ = 1.000	*l* = −15→18
6591 measured reflections	

### Refinement

**Table:**

Refinement on *F*^2^	0 restraints
Least-squares matrix: full	Hydrogen site location: inferred from neighbouring sites
*R*[*F*^2^ > 2σ(*F*^2^)] = 0.048	H-atom parameters constrained
*wR*(*F*^2^) = 0.136	*w* = 1/[σ^2^(*F*_o_^2^) + (0.0703*P*)^2^ + 0.2833*P*] where *P* = (*F*_o_^2^ + 2*F*_c_^2^)/3
*S* = 1.02	(Δ/σ)_max_ < 0.001
3859 reflections	Δρ_max_ = 0.25 e Å^−^^3^
274 parameters	Δρ_min_ = −0.25 e Å^−^^3^

### Special details

**Table:**

Geometry. Bond distances, angles *etc*. have been calculated using the rounded fractional coordinates. All su's are estimated from the variances of the (full) variance-covariance matrix. The cell e.s.d.'s are taken into account in the estimation of distances, angles and torsion angles
Refinement. Refinement on *F*^2^ for ALL reflections except those flagged by the user for potential systematic errors. Weighted *R*-factors *wR* and all goodnesses of fit *S* are based on *F*^2^, conventional *R*-factors *R* are based on *F*, with *F* set to zero for negative *F*^2^. The observed criterion of *F*^2^ > σ(*F*^2^) is used only for calculating -*R*-factor-obs *etc*. and is not relevant to the choice of reflections for refinement. *R*-factors based on *F*^2^ are statistically about twice as large as those based on *F*, and *R*-factors based on ALL data will be even larger.

### Fractional atomic coordinates and isotropic or equivalent isotropic displacement parameters (Å^2^)

**Table:**

	*x*	*y*	*z*	*U*_iso_*/*U*_eq_	
O1	0.8499 (3)	1.73494 (13)	0.98882 (10)	0.0517 (5)	
O2	0.8373 (2)	1.64288 (12)	1.10828 (8)	0.0388 (4)	
O3	0.02746 (18)	1.11133 (11)	0.77597 (8)	0.0320 (3)	
N1	0.6209 (2)	1.07389 (14)	0.70106 (10)	0.0308 (4)	
N2	0.3691 (2)	0.99670 (14)	0.77918 (10)	0.0303 (4)	
C1	0.5243 (2)	1.10427 (16)	0.77304 (11)	0.0296 (5)	
C2	0.3686 (3)	0.89049 (17)	0.70448 (12)	0.0307 (5)	
C3	0.5245 (3)	0.93989 (17)	0.65689 (12)	0.0304 (5)	
C4	0.5945 (3)	0.87422 (17)	0.57186 (12)	0.0323 (5)	
C5	0.5483 (3)	0.73492 (19)	0.53904 (14)	0.0420 (6)	
C6	0.6122 (3)	0.6785 (2)	0.45708 (15)	0.0480 (6)	
C7	0.7243 (3)	0.7582 (2)	0.40643 (13)	0.0441 (6)	
C8	0.7719 (3)	0.8962 (2)	0.43826 (13)	0.0421 (6)	
C9	0.7082 (3)	0.95396 (19)	0.52012 (13)	0.0376 (6)	
C10	0.5889 (2)	1.23891 (17)	0.83951 (12)	0.0306 (5)	
C11	0.6297 (3)	1.35063 (18)	0.80399 (12)	0.0355 (5)	
C12	0.7025 (3)	1.47865 (18)	0.86269 (12)	0.0357 (5)	
C13	0.7361 (2)	1.49682 (16)	0.95828 (12)	0.0303 (5)	
C14	0.6976 (3)	1.38537 (18)	0.99390 (12)	0.0338 (5)	
C15	0.6249 (3)	1.25733 (17)	0.93528 (12)	0.0338 (5)	
C16	0.8137 (3)	1.63675 (17)	1.01870 (12)	0.0337 (5)	
C17	0.2157 (3)	0.99226 (18)	0.84355 (12)	0.0335 (5)	
C18	0.0062 (3)	0.98690 (17)	0.80001 (13)	0.0332 (5)	
C19	−0.1480 (3)	0.9614 (2)	0.86693 (15)	0.0434 (6)	
C20	0.2276 (3)	0.75300 (17)	0.69024 (12)	0.0333 (5)	
C21	0.0656 (3)	0.70164 (19)	0.61958 (14)	0.0434 (6)	
C22	−0.0584 (3)	0.5708 (2)	0.60422 (17)	0.0544 (7)	
C23	−0.0218 (4)	0.4910 (2)	0.65886 (17)	0.0563 (7)	
C24	0.1364 (4)	0.5410 (2)	0.72990 (16)	0.0581 (8)	
C25	0.2614 (4)	0.6721 (2)	0.74577 (14)	0.0465 (6)	
H2	0.88300	1.72160	1.13820	0.0580*	
H3	−0.08050	1.10880	0.74830	0.0480*	
H5	0.47390	0.67970	0.57260	0.0500*	
H6	0.57920	0.58550	0.43580	0.0580*	
H7	0.76740	0.71960	0.35150	0.0530*	
H8	0.84730	0.95070	0.40450	0.0500*	
H9	0.74160	1.04700	0.54090	0.0450*	
H11	0.60790	1.33940	0.74010	0.0430*	
H12	0.72910	1.55290	0.83810	0.0430*	
H14	0.72080	1.39670	1.05770	0.0410*	
H15	0.60000	1.18320	0.95980	0.0410*	
H17A	0.26330	1.07110	0.89580	0.0400*	
H17B	0.20180	0.91390	0.86740	0.0400*	
H18	−0.03550	0.91320	0.74320	0.0400*	
H19A	−0.10920	1.03390	0.92250	0.0650*	
H19B	−0.15270	0.87860	0.88230	0.0650*	
H19C	−0.28010	0.95570	0.83860	0.0650*	
H21	0.04000	0.75520	0.58230	0.0520*	
H22	−0.16690	0.53690	0.55670	0.0650*	
H23	−0.10430	0.40290	0.64770	0.0680*	
H24	0.15990	0.48720	0.76740	0.0700*	
H25	0.36850	0.70580	0.79400	0.0560*	

### Atomic displacement parameters (Å^2^)

**Table:**

	*U*^11^	*U*^22^	*U*^33^	*U*^12^	*U*^13^	*U*^23^
O1	0.0798 (11)	0.0273 (7)	0.0409 (8)	0.0080 (7)	−0.0013 (7)	0.0064 (6)
O2	0.0508 (8)	0.0250 (6)	0.0327 (7)	0.0062 (6)	−0.0012 (6)	−0.0002 (5)
O3	0.0303 (6)	0.0279 (6)	0.0335 (6)	0.0054 (5)	−0.0004 (5)	0.0044 (5)
N1	0.0295 (7)	0.0261 (7)	0.0316 (7)	0.0062 (6)	−0.0012 (6)	0.0011 (6)
N2	0.0271 (7)	0.0277 (7)	0.0318 (7)	0.0083 (6)	−0.0001 (6)	0.0004 (6)
C1	0.0285 (8)	0.0264 (8)	0.0301 (8)	0.0086 (7)	−0.0027 (6)	0.0010 (7)
C2	0.0289 (8)	0.0272 (8)	0.0316 (8)	0.0089 (7)	−0.0031 (7)	0.0000 (7)
C3	0.0295 (8)	0.0278 (8)	0.0300 (8)	0.0087 (7)	−0.0036 (7)	0.0013 (7)
C4	0.0300 (8)	0.0329 (9)	0.0308 (8)	0.0104 (7)	−0.0017 (7)	0.0019 (7)
C5	0.0448 (11)	0.0330 (10)	0.0435 (10)	0.0099 (8)	0.0077 (8)	0.0023 (8)
C6	0.0527 (12)	0.0353 (10)	0.0472 (11)	0.0131 (9)	0.0046 (9)	−0.0060 (9)
C7	0.0427 (11)	0.0521 (12)	0.0329 (9)	0.0197 (9)	0.0014 (8)	−0.0039 (8)
C8	0.0394 (10)	0.0502 (12)	0.0367 (10)	0.0144 (9)	0.0055 (8)	0.0098 (8)
C9	0.0375 (10)	0.0352 (10)	0.0375 (10)	0.0113 (8)	0.0022 (8)	0.0042 (8)
C10	0.0252 (8)	0.0271 (8)	0.0337 (9)	0.0069 (6)	−0.0005 (6)	−0.0012 (7)
C11	0.0378 (9)	0.0335 (9)	0.0293 (9)	0.0071 (7)	0.0005 (7)	0.0020 (7)
C12	0.0403 (10)	0.0277 (9)	0.0361 (9)	0.0063 (7)	0.0019 (7)	0.0078 (7)
C13	0.0275 (8)	0.0268 (8)	0.0327 (9)	0.0078 (7)	0.0008 (7)	0.0012 (7)
C14	0.0370 (9)	0.0318 (9)	0.0284 (8)	0.0092 (7)	−0.0004 (7)	0.0018 (7)
C15	0.0371 (9)	0.0257 (8)	0.0353 (9)	0.0074 (7)	−0.0001 (7)	0.0047 (7)
C16	0.0352 (9)	0.0282 (9)	0.0343 (9)	0.0086 (7)	0.0011 (7)	0.0031 (7)
C17	0.0334 (9)	0.0302 (9)	0.0341 (9)	0.0094 (7)	0.0044 (7)	0.0028 (7)
C18	0.0313 (9)	0.0247 (8)	0.0401 (9)	0.0073 (7)	0.0018 (7)	0.0034 (7)
C19	0.0382 (10)	0.0434 (11)	0.0510 (12)	0.0111 (8)	0.0069 (9)	0.0174 (9)
C20	0.0344 (9)	0.0275 (8)	0.0336 (9)	0.0078 (7)	0.0063 (7)	0.0009 (7)
C21	0.0417 (11)	0.0324 (10)	0.0481 (11)	0.0083 (8)	−0.0020 (9)	−0.0002 (8)
C22	0.0411 (11)	0.0385 (11)	0.0647 (14)	−0.0005 (9)	0.0001 (10)	−0.0065 (10)
C23	0.0623 (14)	0.0296 (10)	0.0615 (14)	−0.0034 (9)	0.0226 (11)	−0.0010 (10)
C24	0.0905 (18)	0.0328 (11)	0.0473 (12)	0.0096 (11)	0.0154 (12)	0.0118 (9)
C25	0.0608 (13)	0.0348 (10)	0.0378 (10)	0.0094 (9)	0.0005 (9)	0.0047 (8)

### Geometric parameters (Å, º)

**Table:**

O1—C16	1.207 (2)	C18—C19	1.509 (3)
O2—C16	1.321 (2)	C20—C25	1.385 (3)
O3—C18	1.433 (2)	C20—C21	1.385 (3)
N1—C1	1.316 (2)	C21—C22	1.386 (3)
N1—C3	1.387 (2)	C22—C23	1.372 (3)
N2—C1	1.365 (2)	C23—C24	1.372 (4)
N2—C2	1.393 (2)	C24—C25	1.389 (3)
N2—C17	1.462 (2)	C5—H5	0.9300
O2—H2	0.8200	C6—H6	0.9300
O3—H3	0.8200	C7—H7	0.9300
C1—C10	1.481 (2)	C8—H8	0.9300
C2—C20	1.485 (3)	C9—H9	0.9300
C2—C3	1.371 (3)	C11—H11	0.9300
C3—C4	1.472 (3)	C12—H12	0.9300
C4—C9	1.395 (3)	C14—H14	0.9300
C4—C5	1.397 (3)	C15—H15	0.9300
C5—C6	1.382 (3)	C17—H17A	0.9700
C6—C7	1.378 (3)	C17—H17B	0.9700
C7—C8	1.382 (3)	C18—H18	0.9800
C8—C9	1.385 (3)	C19—H19A	0.9600
C10—C15	1.394 (2)	C19—H19B	0.9600
C10—C11	1.387 (3)	C19—H19C	0.9600
C11—C12	1.383 (3)	C21—H21	0.9300
C12—C13	1.391 (2)	C22—H22	0.9300
C13—C14	1.386 (3)	C23—H23	0.9300
C13—C16	1.491 (2)	C24—H24	0.9300
C14—C15	1.383 (3)	C25—H25	0.9300
C17—C18	1.528 (3)		
			
C1—N1—C3	106.24 (15)	C22—C23—C24	120.1 (2)
C1—N2—C2	106.50 (14)	C23—C24—C25	120.0 (2)
C1—N2—C17	128.48 (15)	C20—C25—C24	120.4 (2)
C2—N2—C17	124.71 (15)	C4—C5—H5	120.00
C16—O2—H2	109.00	C6—C5—H5	120.00
C18—O3—H3	109.00	C5—C6—H6	120.00
N1—C1—N2	111.74 (15)	C7—C6—H6	120.00
N1—C1—C10	122.02 (15)	C6—C7—H7	120.00
N2—C1—C10	126.19 (14)	C8—C7—H7	120.00
N2—C2—C3	106.17 (16)	C7—C8—H8	120.00
C3—C2—C20	131.12 (17)	C9—C8—H8	120.00
N2—C2—C20	122.60 (16)	C4—C9—H9	120.00
N1—C3—C4	120.13 (17)	C8—C9—H9	120.00
N1—C3—C2	109.35 (16)	C10—C11—H11	120.00
C2—C3—C4	130.52 (17)	C12—C11—H11	120.00
C3—C4—C9	118.87 (16)	C11—C12—H12	120.00
C3—C4—C5	123.02 (17)	C13—C12—H12	120.00
C5—C4—C9	118.10 (17)	C13—C14—H14	120.00
C4—C5—C6	120.58 (19)	C15—C14—H14	120.00
C5—C6—C7	120.9 (2)	C10—C15—H15	120.00
C6—C7—C8	119.21 (19)	C14—C15—H15	120.00
C7—C8—C9	120.50 (19)	N2—C17—H17A	109.00
C4—C9—C8	120.76 (18)	N2—C17—H17B	109.00
C11—C10—C15	119.05 (16)	C18—C17—H17A	109.00
C1—C10—C11	118.17 (15)	C18—C17—H17B	109.00
C1—C10—C15	122.60 (16)	H17A—C17—H17B	108.00
C10—C11—C12	120.58 (16)	O3—C18—H18	109.00
C11—C12—C13	120.27 (17)	C17—C18—H18	109.00
C12—C13—C16	118.27 (16)	C19—C18—H18	109.00
C12—C13—C14	119.29 (16)	C18—C19—H19A	109.00
C14—C13—C16	122.44 (16)	C18—C19—H19B	109.00
C13—C14—C15	120.49 (16)	C18—C19—H19C	109.00
C10—C15—C14	120.32 (17)	H19A—C19—H19B	109.00
O2—C16—C13	113.42 (15)	H19A—C19—H19C	109.00
O1—C16—C13	123.33 (16)	H19B—C19—H19C	110.00
O1—C16—O2	123.25 (17)	C20—C21—H21	120.00
N2—C17—C18	113.88 (14)	C22—C21—H21	120.00
O3—C18—C17	107.26 (15)	C21—C22—H22	120.00
O3—C18—C19	112.97 (16)	C23—C22—H22	120.00
C17—C18—C19	109.78 (16)	C22—C23—H23	120.00
C2—C20—C21	120.99 (17)	C24—C23—H23	120.00
C21—C20—C25	118.92 (18)	C23—C24—H24	120.00
C2—C20—C25	120.05 (18)	C25—C24—H24	120.00
C20—C21—C22	120.29 (19)	C20—C25—H25	120.00
C21—C22—C23	120.3 (2)	C24—C25—H25	120.00
			
C3—N1—C1—N2	0.37 (19)	C5—C4—C9—C8	0.4 (3)
C3—N1—C1—C10	177.80 (15)	C3—C4—C5—C6	177.8 (2)
C1—N1—C3—C4	179.15 (17)	C9—C4—C5—C6	−0.7 (3)
C1—N1—C3—C2	−0.4 (2)	C4—C5—C6—C7	0.6 (3)
C17—N2—C1—N1	−173.96 (16)	C5—C6—C7—C8	−0.3 (3)
C2—N2—C1—N1	−0.23 (19)	C6—C7—C8—C9	0.1 (3)
C17—N2—C2—C20	−9.4 (3)	C7—C8—C9—C4	−0.2 (3)
C2—N2—C1—C10	−177.53 (16)	C1—C10—C15—C14	175.83 (17)
C17—N2—C1—C10	8.7 (3)	C11—C10—C15—C14	0.9 (3)
C1—N2—C2—C20	176.53 (17)	C15—C10—C11—C12	−0.9 (3)
C1—N2—C2—C3	0.0 (2)	C1—C10—C11—C12	−176.03 (17)
C17—N2—C2—C3	174.02 (17)	C10—C11—C12—C13	0.1 (3)
C2—N2—C17—C18	−69.5 (2)	C11—C12—C13—C16	−179.71 (18)
C1—N2—C17—C18	103.2 (2)	C11—C12—C13—C14	0.6 (3)
N2—C1—C10—C15	49.3 (2)	C12—C13—C16—O1	−0.4 (3)
N1—C1—C10—C11	47.3 (2)	C12—C13—C16—O2	179.00 (17)
N1—C1—C10—C15	−127.74 (19)	C14—C13—C16—O1	179.3 (2)
N2—C1—C10—C11	−135.71 (18)	C14—C13—C16—O2	−1.3 (3)
N2—C2—C3—N1	0.2 (2)	C12—C13—C14—C15	−0.5 (3)
N2—C2—C20—C21	108.0 (2)	C16—C13—C14—C15	179.75 (18)
C20—C2—C3—C4	4.7 (4)	C13—C14—C15—C10	−0.2 (3)
C3—C2—C20—C25	101.1 (3)	N2—C17—C18—O3	−65.48 (19)
N2—C2—C3—C4	−179.22 (19)	N2—C17—C18—C19	171.44 (16)
C20—C2—C3—N1	−175.90 (19)	C2—C20—C21—C22	176.72 (19)
N2—C2—C20—C25	−74.5 (3)	C25—C20—C21—C22	−0.9 (3)
C3—C2—C20—C21	−76.4 (3)	C2—C20—C25—C24	−176.7 (2)
N1—C3—C4—C5	160.89 (19)	C21—C20—C25—C24	0.9 (3)
C2—C3—C4—C9	158.7 (2)	C20—C21—C22—C23	0.0 (3)
N1—C3—C4—C9	−20.7 (3)	C21—C22—C23—C24	0.9 (4)
C2—C3—C4—C5	−19.7 (3)	C22—C23—C24—C25	−0.9 (4)
C3—C4—C9—C8	−178.07 (19)	C23—C24—C25—C20	0.0 (4)

### Hydrogen-bond geometry (Å, º)

Cg1 is the centroid of the N1/N2/C1–C3 ring.

**Table:**

*D*—H···*A*	*D*—H	H···*A*	*D*···*A*	*D*—H···*A*
O2—H2···O3^i^	0.82	1.86	2.6718 (17)	170
O3—H3···N1^ii^	0.82	2.04	2.8377 (19)	166
C19—H19*B*···O1^iii^	0.96	2.46	3.355 (3)	155
C24—H24···O2^iv^	0.93	2.57	3.482 (3)	167
C19—H19*C*···*Cg*1^ii^	0.96	2.53	3.422 (2)	154

Symmetry codes: (i) −*x*+1, −*y*+3, −*z*+2; (ii) *x*−1, *y*, *z*; (iii) *x*−1, *y*−1, *z*; (iv) −*x*+1, −*y*+2, −*z*+2.
