# Supplementary figures and images for: Crystal structure of 4-[1-(2-hy­droxy­prop­yl)-4,5-diphenyl-1H-imidazol-2-yl]benzoic acid
Source: Acta Crystallogr E Crystallogr Commun. 2015 Jan 3;71(Pt 2):o77–8. doi: 10.1107/S2056989014027078 (PMC4384621; doi:10.1107/S2056989014027078)

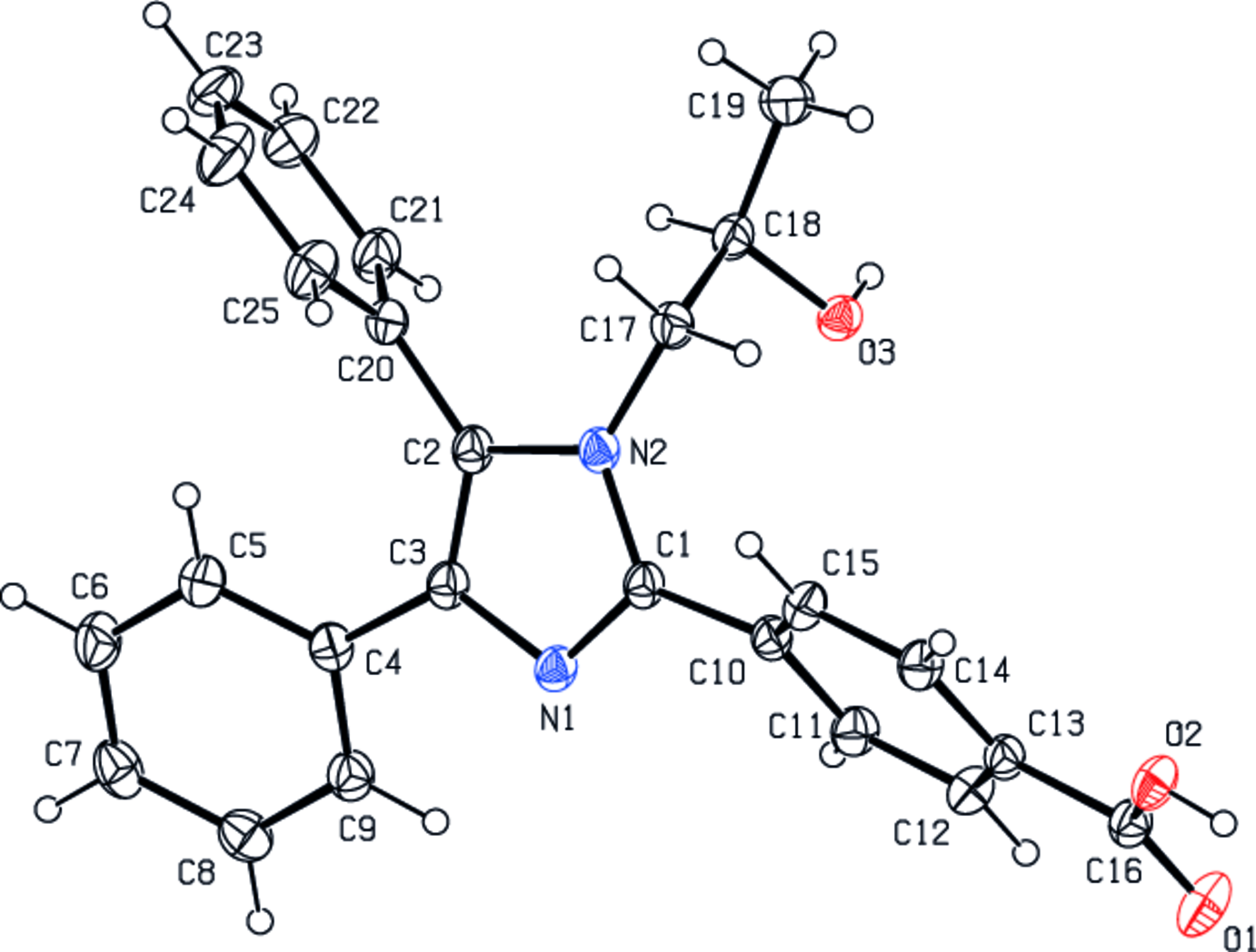

Supplement: Supplementary file 4 [file e-71-00o77-fig1.tif]

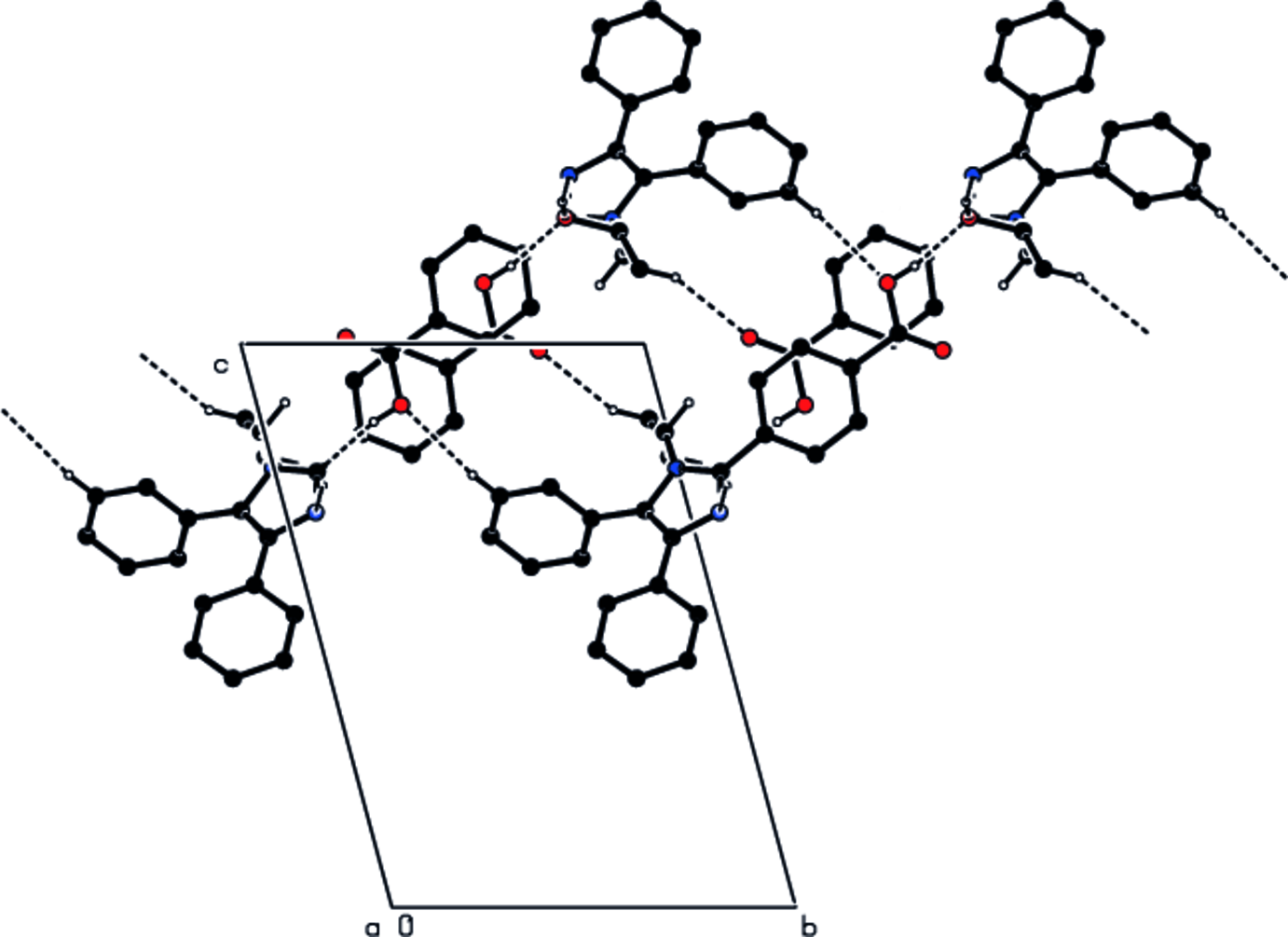

Supplement: Supplementary file 5 [file e-71-00o77-fig2.tif]
